# Musculoskeletal modelling of muscle activation and applied external forces for the correction of scoliosis

**DOI:** 10.1186/1743-0003-11-52

**Published:** 2014-04-07

**Authors:** Maurice Curtin, Madeleine M Lowery

**Affiliations:** 1 School of Electrical, Electronic and Communications Engineering, University College Dublin, Dublin, Belfield, Ireland

**Keywords:** Muscle activation, Electrical stimulation, Scoliosis, Optimization, Biomechanical modelling

## Abstract

**Background:**

This study uses biomechanical modelling and computational optimization to investigate muscle activation in combination with applied external forces as a treatment for scoliosis. Bracing, which incorporates applied external forces, is the most popular non surgical treatment for scoliosis. Non surgical treatments which make use of muscle activation include electrical stimulation, postural control, and therapeutic exercises. Electrical stimulation has been largely dismissed as a viable treatment for scoliosis, although previous studies have suggested that it can potentially deliver similarly effective corrective forces to the spine as bracing.

**Methods:**

The potential of muscle activation for scoliosis correction was investigated over different curvatures both with and without the addition of externally applied forces. The five King’s classifications of scoliosis were investigated over a range of Cobb angles. A biomechanical model of the spine was used to represent various scoliotic curvatures. Optimization was applied to the model to reduce the curves using combinations of both deep and superficial muscle activation and applied external forces.

**Results:**

Simulating applied external forces in combination with muscle activation at low Cobb angles (< 20 degrees) over the 5 King’s classifications, it was possible to reduce the magnitude of the curve by up to 85% for classification 4, 75% for classifications 3 and 5, 65% for classification 2, and 60% for classification 1. The reduction in curvature was less at larger Cobb angles. For King’s classifications 1 and 2, the serratus, latissimus dorsi, and trapezius muscles were consistently recruited by the optimization algorithm for activation across all Cobb angles. When muscle activation and external forces were applied in combination, lower levels of muscle activation or less external force was required to reduce the curvature of the spine, when compared with either muscle activation or external force applied in isolation.

**Conclusions:**

The results of this study suggest that activation of superficial and deep muscles may be effective in reducing spinal curvature at low Cobb angles when muscle groups are selected for activation based on the curve type. The findings further suggest the potential for a hybrid treatment involving combined muscle activation and applied external forces at larger Cobb angles.

## Background

**Figure 1:**
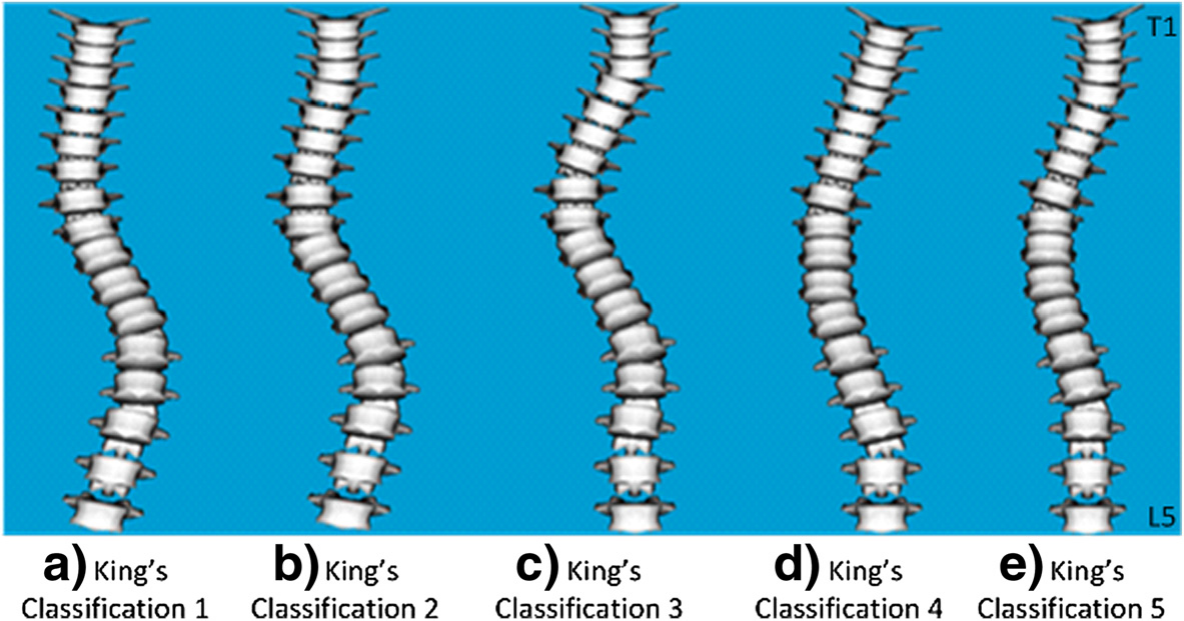
**The five King’s classifications of scoliotic curve **[3]**, illustrated from T1 to L5. a)** Classification 1: Double curve of the thoracic and lumbar spine. **b)** Classificiation 2: Double curve of the thoracic and the lumbar spine with less prominent lumbar curvature. **c)**  Classification 3: Single primary thoracic curve. **d)** Classification 4: Long thoracic curve. **e)** Classification 5: Double thoracic curve. Illustrated using OpenSim [33].

Idiopathic scoliosis effects approximately 3% of children and adolescents [1] and is defined as a lateral curvature of the spine with rotation of the vertebrae within the curve. Scoliosis is traditionally evaluated using the Cobb angle, measured between the intersection of the lines tangential to the vertebral endplates which make up the lowermost and uppermost parts of the scoliotic curve. The presence of scoliosis is typically defined for Cobb angles greater than 10 degrees. The scoliotic curve may be classified using the widely accepted King’s classification scheme which classifies curves into one of five categories, based on the location and shape of the curve on the spine [2], Figure 1. Scoliosis can be treated both surgically and non-surgically. Surgical treatment involves the application of rods and hooks to the vertebrae and the spine is then pushed and fused into place. This generally restricts the overall movement of the spine, but is necessary in extreme cases of scoliosis. An alternative is non-surgical treatment which takes place over a number of years. Bracing is currently the most popular of non-surgical treatment methods for scoliosis [3,4]. Alternative non-surgical methods have made use of muscle activation patterns for scoliosis correction and include electrical stimulation [5-7], therapeutic exercise [8] and postural control [9]. All of the above methods have demonstrated varied levels of success in controlling the progression of scoliotic curves. Electrical stimulation, in particular, has had limited success. A prospective study by Nachemson *et al.* comparing the effectiveness of bracing and stimulation for the correction of scoliosis concluded that electrical stimulation is not an effective treatment [10]. However, variation in electrode placements and stimulation details, such as the duration of the applied stimulation, were not considered. Furthermore, the Cobb angles included in that study were restricted to a minimum of 25°, with lower Cobb angles not considered. A similar study by Rowe *et al.*[11] reported a weighted mean success rate of 0.39 with muscle stimulation for the treatment of scoliosis in contrast to rates of up to 0.93 with bracing. A more recent *in vivo* study has hinted at reviving stimulation treatment for scoliosis [12]. The results of that study suggest that that electrical stimulation may be effective in correcting scoliotic curvature, particularly at Cobb angles of 20° or less. Specific therapeutic exercises developed for scoliosis correction which also elicit muscle activation have demonstrated success in reducing the progression rate of scoliotic curvature and reducing the magnitude of the Cobb angle [8]. These may provide an alternative non-surgical method to limit the progression of spinal curvature using muscle activation.

Biomechanical models of the spine have been used for several decades to investigate muscle and brace force patterns and their effects on scoliotic curves [13-21]. It has been hypothesised that forces which deliver small initial correction will achieve a larger correction in the long term with continuing treatment [22]. Computational studies for scoliosis correction have, therefore, assumed a force pattern which will deliver the best possible immediate correction to the curve [17,20]. The majority of modelling studies which have considered treatments for scoliosis have focused on bracing and surgery [20,23,24]. To examine the corrective potential of muscle forces on a scoliotic curve, Wynarsky *et al.*[17] applied an optimization to a computer model of a curve, comparing simulations of muscle forces and brace forces. It was suggested that within the defined constraints, muscle activation is more effective at correcting the curve than passive brace forces. However it was also noted that it was not possible to reproduce the specific optimal muscle activation patterns *in vivo*. The conclusion of the modelling study presented in [17] is in contrast to the findings of [10], and poses the question as to whether muscle forces elicited by electrical stimulation or targeted physical therapy could potentially deliver a more effective corrective treatment than can be achieved by bracing or postural control alone.

The study presented in this paper addresses this question across a range of spinal configurations using the concept of generalised external forces in place of forces specific to a particular brace type. There has not been much progress in the field of non-surgical scoliosis correction in many years. With the advance in electrode technology in recent years, targeted stimulation of superficial and deep muscles is now possible through the use of implantable electrodes [25]. Muscle activation may thus have potential in scoliosis correction that has not yet been explored. Both superficial and deep muscle groups were, therefore, analysed in this study.

The aim of this study was to investigate the potential of deep and superficial muscle activation in combination with applied external forces, for the correction of scoliosis in a computer model. Previous studies have shown that lateral placement of electrodes on the convex side of the curve delivers the best results using surface electrode stimulation [5]. The results obtained using the model are tested against these guidelines to examine whether a theoretically more effective electrode placement exists and if so, whether it varies across all of the five King’s classifications. These curve classifications were chosen to avoid limiting the study to one typical scoliotic curve type and to examine how the muscle activation patterns adapted across the different curvatures.

## Methods

A model of the thoracolumbar spine, ribcage and sternum incorporating deep and superficial muscles of the lumbar and thoracic regions was constructed in Matlab (Mathworks, MA, USA). The five King’s classifications [2] were simulated by adjusting the vertebral positions relative to one another. For each classification, the Cobb angle was adjusted across the range 10° – 60°. In King’s classifications 1 and 2, curves are present in both the lumbar and thoracic areas, the Cobb measurement was thus based on the lumbar curve in these cases, and the thoracic curve was adjusted proportionally. The model inputs were the forces elicited from muscle activation patterns and applied external forces. The outputs of the model were the resultant distances moved by each individual body in the model.

The deep and superficial muscle architecture of the lumbar and abdominal regions was included as described by Stokes and Morse [26]. The multifidus muscles were excluded as they are unlikely to deliver corrective forces to the spine due to their location and orientation. The thoracic muscle architecture was based on that described in [16]. Muscles were classified as being either ‘superficial’ or ‘deep’ according to their accessibility for electrical stimulation. Although the focus was not exclusively on muscle activation as a result of electrical stimulation, this was deemed the most suitable method to classify the muscle groups. The superficial muscles consisted of the latissimus dorsi, trapezius, rectus abdominis, serratus, pectorals and external abdominals. The deep muscles were identified as the psoas, intercostals, erector spinae, quadratus lumborum and internal abdominals.

Global vertebral positions of the model were based on a cadaver study [18], while all remaining joint stiffness and body positions were based on the model presented by Takashima *et al.*[16]. Gravity was omitted from the model, as the subject was assumed to be prone. All force displacement relationships were solved using a direct stiffness procedure [14]. The main model parameters are presented in Tables 1 and 2. The model was locked at the sacrum to ensure no displacement below the lumbosacral joint. Translational displacement of the T1 vertebra was restricted in the *x* (medial/lateral) and *z* (anterior/posterior) directions was restricted to simulate the *in vivo* mechanical restraints of the thorax [20].

**Table 1:** Model positions

	**x**	**y**	**z**
**T1 vertebra**	0	454.9	19.4
**T2 vertebra**	0	437.3	-14.4
**T3 vertebra**	0	418	-20.8
**T4 vertebra**	0	397.2	-23
**T5 vertebra**	0	375.3	-22.8
**T6 vertebra**	0	351.5	-21.5
**T7 vertebra**	0	326.8	-20.4
**T8 vertebra**	0	302.5	-20
**T9 vertebra**	0	278.2	-20.2
**T10 vertebra**	0	252.8	-20.4
**T11 vertebra**	0	225.1	-19.5
**T12 vertebra**	0	196	-15.8
**L1 vertebra**	0	166.3	-8.4
**L2 vertebra**	0	133.9	3.6
**L3 vertebra**	0	100.6	17.4
**L4 vertebra**	0	64.8	26.2
**L5 vertebra**	0	31.7	16.2
**Left Rib 1**	24.15	437.4	41.65
**Left costo-vertebral attachment T1**	15.15	454.9	29.65
**Left costo-transverse attachment T1**	40	454.6	9.9
**Left Costal-cartilage attachment 1**	30	437.4	41.65

The global positions of the model components [15,26,27]. Values are summarized and data for the left side of the model can be replicated on the right side by inverting the x value. Remaining data can be found in [16,27], and translated based on data presented in this table. Data are presented in mm.

**Table 2:** Model stiffness

	**Tx (N/mm)**	**Ty (N/mm)**	**Tz (N/mm)**	**Rxyz (Nm/deg)**
**T1**	686.7	588.6	196.2	0.3
**T2**	1177.2	1079.1	294.3	0.6
**T4**	2060.1	1863.9	588.6	1.7
**T5**	1863.9	1667.7	588.6	1.7
**T6**	1765.8	1569.6	588.6	1.7
**T7**	1471.5	1373.4	588.6	1.7
**T8**	1471.5	1275.3	588.6	1.8
**T9**	1471.5	1373.4	686.7	1.8
**T10**	1471.5	1373.4	686.7	2.1
**T11**	1471.5	1079.1	784.8	1.7
**T12**	1765.8	981	981	1.5
**L1**	1569.6	882.9	1177.2	1.5
**L3**	1471.5	784.8	1177.2	1.5
**L4**	1373.4	686.7	1079.1	1.3
**L5**	1079.1	588.6	882.9	1.2
**Costo-vertebral**	49.05	49.05	49.05	0.2
**Costo-transverse**	49.05	49.05	49.05	0.2
**Costal cartilage**	73.575	73.575	24.525	0.2

Rotational (Rxyz) and translational (Tx, Ty, Tz) stiffness values for the model components [13,15]. Stiffness values are relative (between adjacent bodies). The global coordinate system was implemented with the positive x-axis (medial/lateral) to the left of the model, the positive y-axis (superior/inferior) facing upwards, and the positive z-axis (anterior/posterior) facing forward.

Three external forces, limited to a maximum of 100 N were available to the model. These were comprised of two thoracic and one lumbar force, as applied in [20]. The thoracic forces were free to act on any of the ten ribs while the lumbar force could act on any one of the five lumbar vertebrae. All forces acted in the transverse plane. The force locations were chosen based on a typical three-point corrective force pattern applied by a brace [28]. The forces included in this study, however, were not intended to be representative of a specific brace and could potentially be delivered *in vivo* through either postural control or with the application of a brace.

For each King’s classification and Cobb angle, an optimization routine was performed to identify the set of corrective forces which reduced the curvature by minimizing the displacement of all vertebrae from the sagittal plane. All muscle groups were made available to the model, both with and without the inclusion of the externally applied forces. The simulations and optimizations were repeated with deep muscle groups excluded from the model. This was done to examine the potential contribution to the overall results by the deep muscle groups which are typically more difficult to isolate.

The optimization was performed using the glcDirect solver developed by Tomlab optimization software (Tomlab, WA, USA) for Matlab (Mathworks, MA, USA). This solver implements an extended version of the DIRECT algorithm developed by Jones *et al*., [29]. The sum of the squares of the distances of the vertebrae from the sagittal plane, *D*, was chosen as the objective function to be minimized,

(1) $$D=\sum_{i=1}^{n}{\left(Vd_{i}-Vn_{i}\right)}^{2}$$

where *Vd*_*i*_ is the global starting position of vertebra *i* and *Vn*_*i*_ is the global position of vertebra *i* in a normal, straight spine. *n* represents the total number of vertebrae. This follows the approach used in previous studies of spinal curvature correction in which through minimisation of the objective function, the scoliotic curve was moved as close towards a normal alignment as possible [17,20]. The initial values for the objective functions are listed in Table 3. Static equilibrium of the model was imposed, following the approach described by Stokes and Gardner-Morse [19],

**Table 3:** Objective functions

	**10°**	**20°**	**30°**	**40°**	**50°**	**60°**
**Classification 1**	265.31	1272.02	2988.77	5346.61	8256.51	11618.49
**Classification 2**	267.73	1268.14	2917.32	5064.02	7522.67	10102.04
**Classification 3**	637.78	2508.58	5487.59	9375.95	13913.97	18798.27
**Classification 4**	735.26	2918.7	6483.99	11322.78	17288.05	24198.57
**Classification 5**	352.27	1397.05	3098.9	5400.69	8226.4	11484.81

The initial objective functions described in Equation 1 for the 5 classifications. Data are presented every 10° for Cobb angles between 10° and 60°. Values are presented in mm^2^.

(2) $$F_{M}+F_{\text{EXT}}-\mathrm{KD}=0$$

where *F*_*M*_ represents muscle forces, *F*_*EXT*_ represents external forces, *K* represents model stiffness, and *D* represents model displacement. All individual model body displacements relative to one another were limited to 5 degrees rotation and 5 mm translation in the sagittal plane, and 2 degrees rotation and 2 mm translation in all other planes following an approach previously used to represent physiological limits [19]; each external force was constrained between 0 N and 100 N and the activation levels of each individual muscle was constrained to a value of either 0 or 1. Partial activation of muscles was not considered in this study to eliminate solutions comprised of complex muscle activation patterns that would be difficult to elicit *in vivo*. This made it possible to identify on key muscle groups that could contribute to the curve correction by delivering substantial forces to the model.

## Results

**Figure 2:**
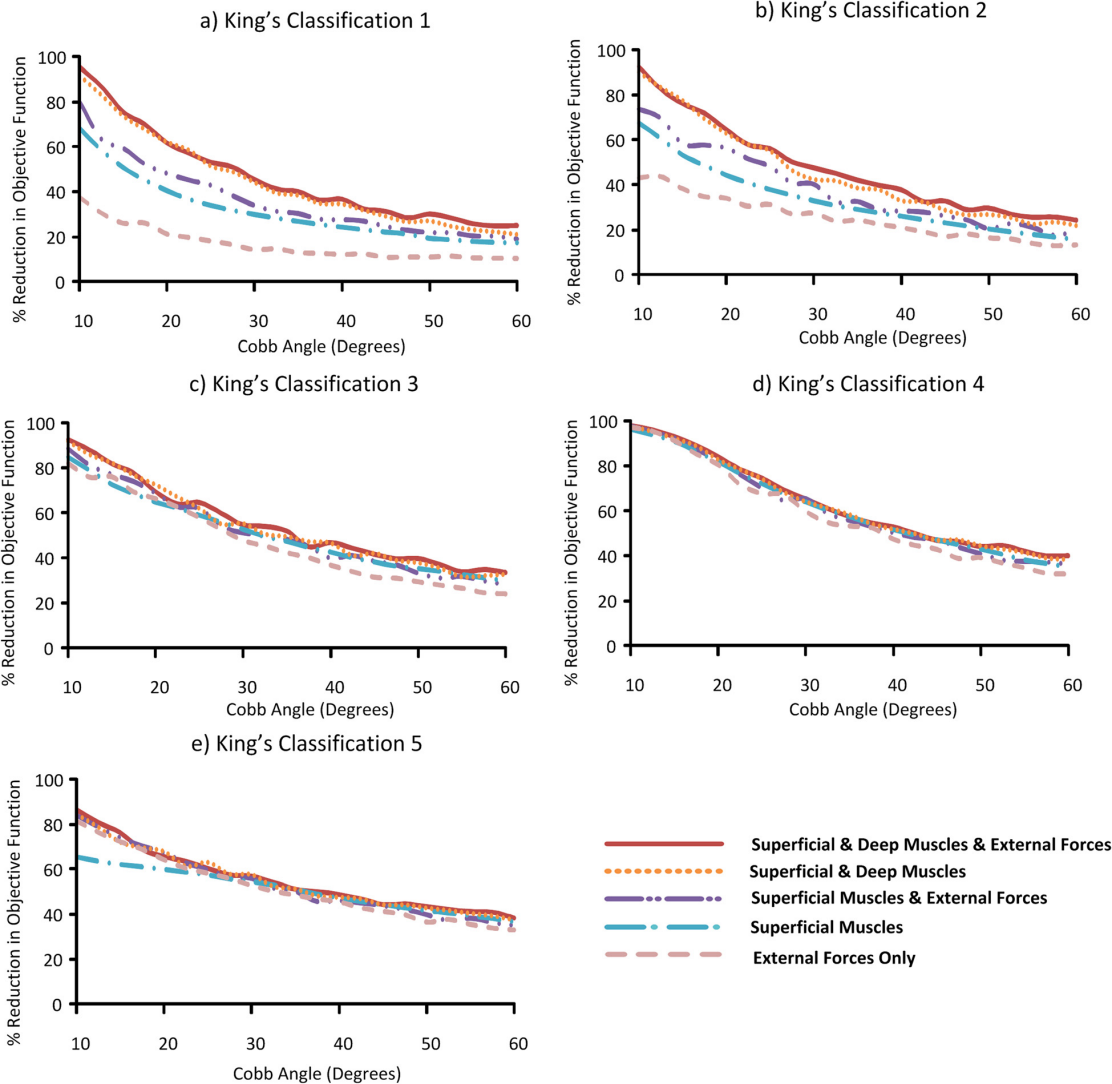
**The percentage reduction in objective function across all Cobb angles for the individual King’s classifications (a-e).** Results are presented when superficial and deep muscles are available to the model, superficial muscles only, both muscle combinations with externally applied forces, and applied external forces only.

The overall curve correction obtained by the optimization algorithm was less for larger values of the Cobb angle. The ability of the applied muscle and external forces to correct the curvature also varied across the five King’s Classifications, reflecting the different morphology of the curves, Figure 2(a-e). Taking the most effective muscle and external force combination, at a Cobb angle of 20°, the objective function was reduced by approximately 85% for classification 4, 75% for classifications 3 and 5, 65% for classification 2, and 60% for classification 1, Figure 2. At a Cobb angle of 40°, the objective function was reduced by approximately 60% for classification 5, 55% for classifications 3 and 4, and 40% for classifications 1 and 2**.** The differences observed in the percentage reduction between classifications increased further when analysing the application of external forces only. The difference was most apparent in classification 1 when comparing the improvement achieved using external forces only, to that achieved using other muscle and force combinations. Compared with muscle and external force combinations, the reduction in objective function was approximately 20% lower using external forces only between Cobb angles of 10° and 30°, Figure 2a. As the Cobb angle increased, the difference between application of external forces alone and the remaining muscle and force combinations was less pronounced.

**Figure 3:**
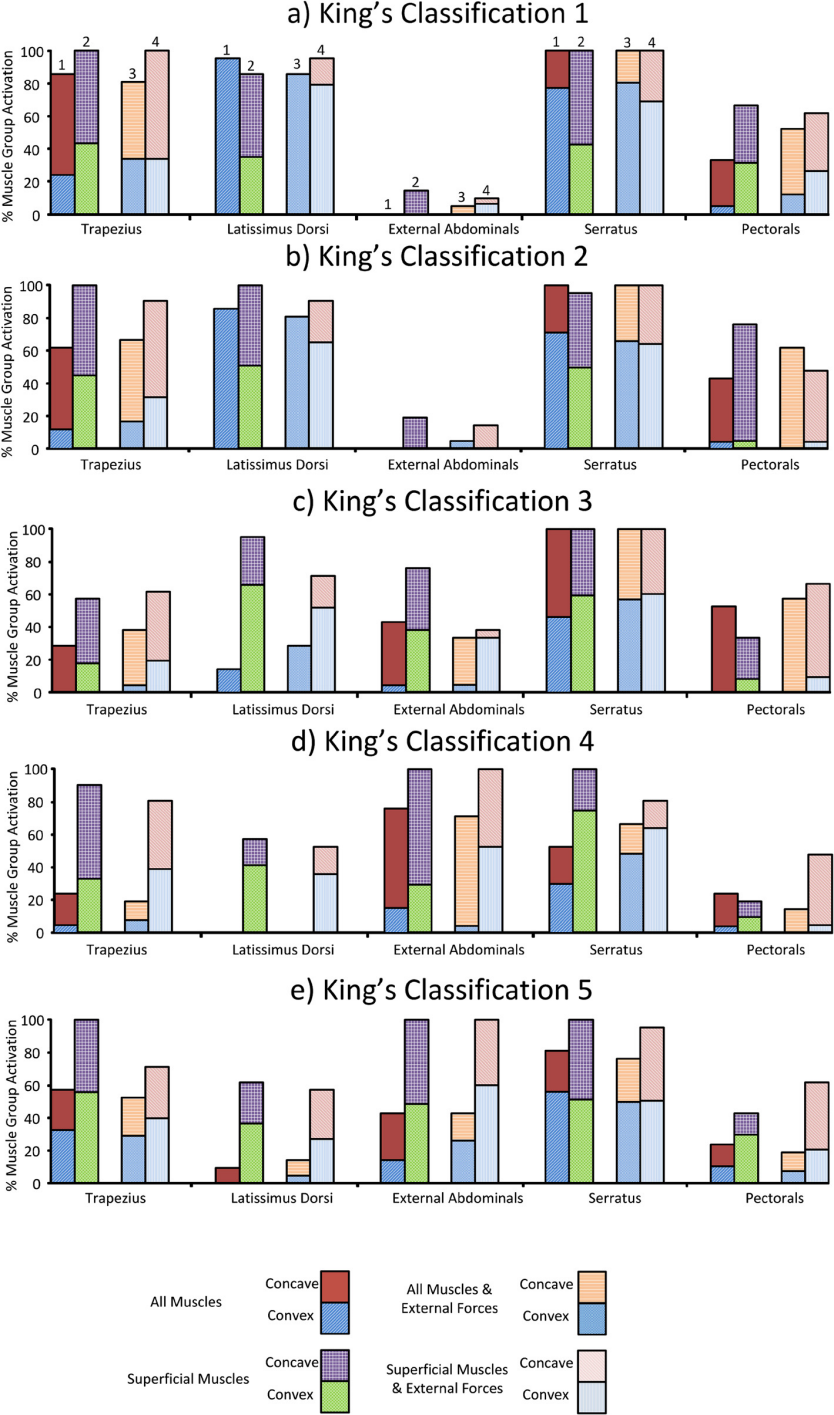
**Superficial Muscles: The percentage of optimizations for which each superficial muscle group was selected for activation for the individual King’s classifications (a-e).** Column 1 illustrates the result for when superficial and deep muscles were included in the model. Column 2 illustrates the result for when only superficial muscles were included in the model. Columns 3 and 4 illustrate the equivalent results with the addition of applied external forces to the model. The graph also indicates which side of the thoracic curve that the muscles were located. The top area of each column represents the concave side, while the bottom area represents the convex side.

A muscle group was considered active if any muscle within that group was activated for a given optimization. For King’s classifications 1 and 2, the serratus, latissimus dorsi, and trapezius, were recruited by the optimization algorithm for activation. The serratus was targeted similarly for classifications 3–5, Figure 3. The rectus abdominis muscle group was omitted from Figure 3 as it was only chosen during a small percentage of optimizations in classification 4 (<5%).

**Figure 4:**
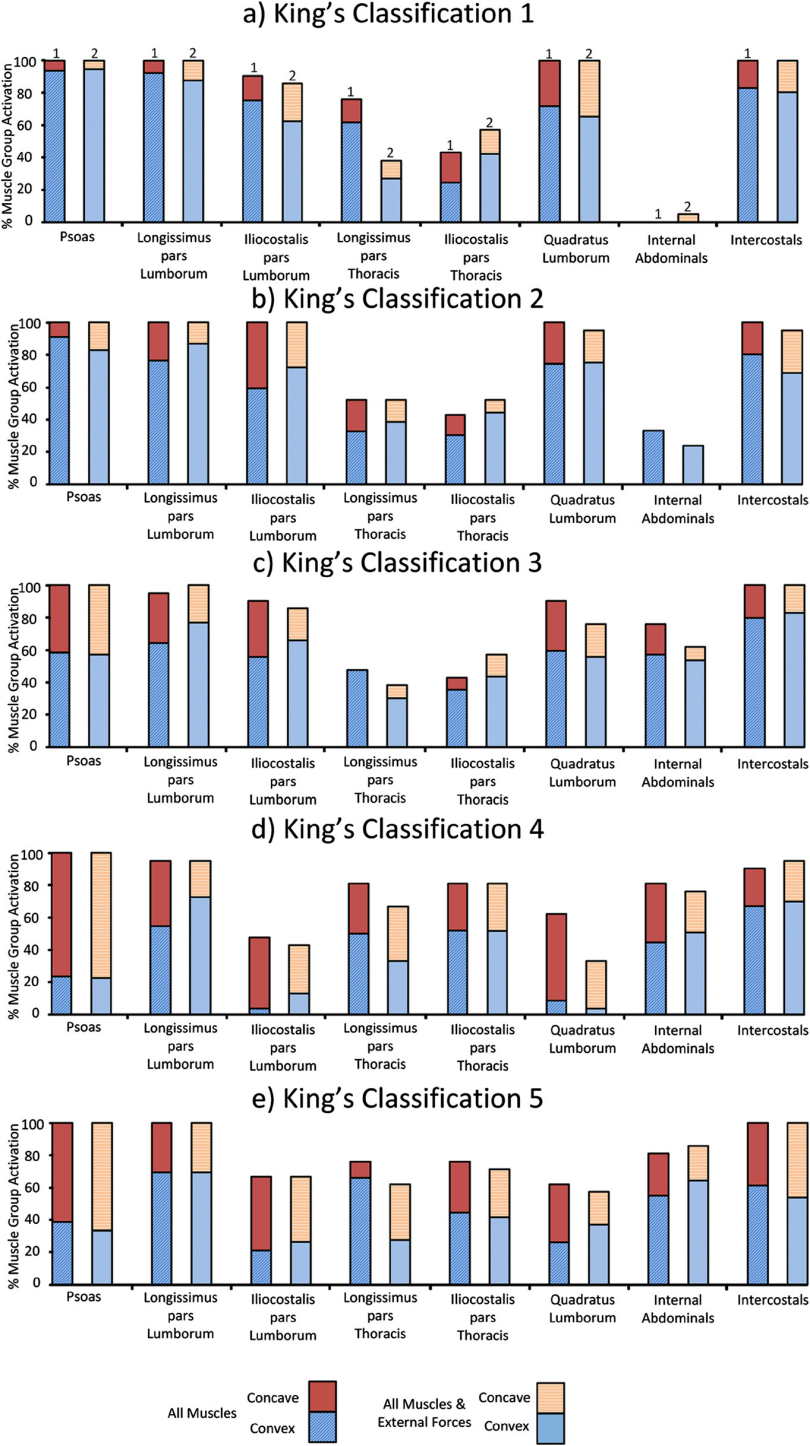
**Deep Muscles: The percentage of optimizations for which each deep muscle group was selected for activation for the individual King’s classifications (a-e).** Column 1 illustrates the result for when superficial and deep muscles were included in the model. Column 2 illustrates the equivalent results with the addition of applied external forces to the model. The graph also indicates which side of the thoracic curve that the muscles were located. The top area of each column represents the concave side, while the bottom area represents the convex side.

When deep muscles were included in the optimisation, the psoas, longissimus pars lumborum and intercostals muscles were consistently selected by the optimization, Figure 4. The distribution of muscles activated on each side of the curve varied with the curve classification. In King’s classifications 1 and 2, deep muscles were predominantly activated on the convex side of the thoracic curve, Figure 4(a,b). This was also true for the majority of superficial muscles when external forces were not included in the optimization, Figure 3(a,b). In classifications 3–5, additional activation of muscles was introduced on the concave side, Figures 3(c-e) and 4(c-e).

**Figure 5:**
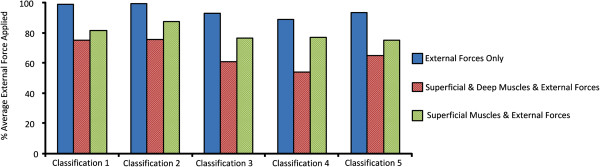
**External forces.** The average of the 3 external forces applied to the model over all Cobb angles for the five classifications.

**Figure 6:**
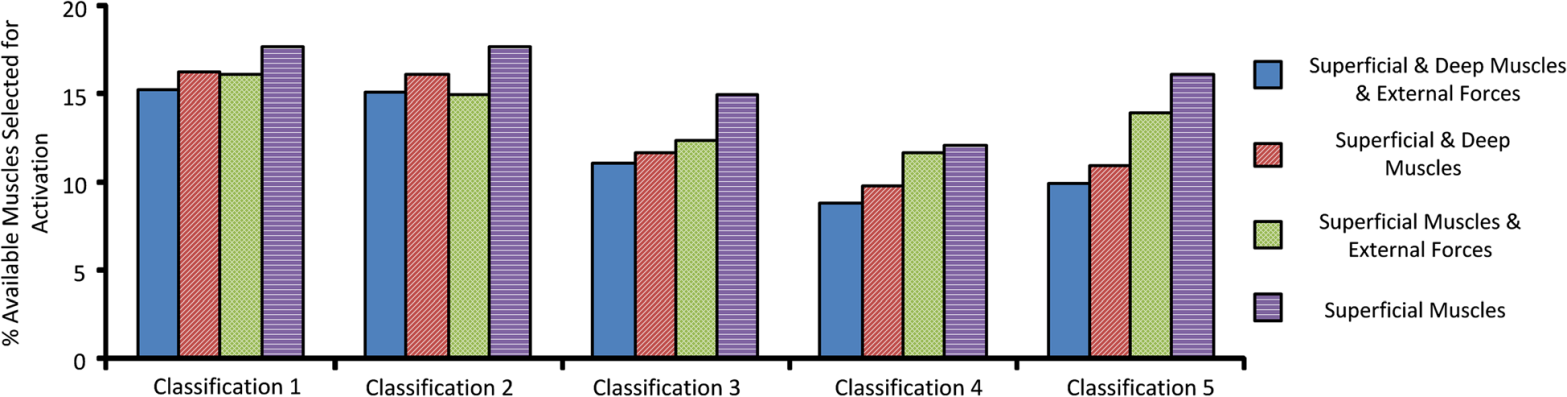
**Muscle activation.** The percentage of available muscles selected for activation over all Cobb angles for the five classifications.

The optimization algorithm consistently identified the largest average external forces when muscles were excluded from the optimization, Figure 5. Similarly, the greatest percentage of muscles activated across all classifications occurred when external forces were excluded from the optimization, Figure 6. When both muscle forces and applied external forces were available to the optimization algorithm, the lowest external forces were required when both superficial and deep muscles were included in the optimization. The average reduction in external force when muscles were also included in the optimization ranged between 25-35% of the maximum average external force applied, Figure 5. This averages over all 3 external forces, representing a reduction of up to 90 N in total in some cases. The corrective external forces identified by the optimisation algorithm for each model are summarized in Table 4.

**Table 4:** Applied external forces

**Classification 1**	**All muscles**		**Superficial muscles**		**No muscles**	
Left	58.22	(23.5)	77.26	(16.18)	97.67	(6.07)
Right	78.9	(13.11)	80.06	(23.79)	99.98	(0.05)
Lumbar	87.59	(12.23)	86.5	(11.83)	99.27	(1.17)
**Classification 2**	**All muscles**		**Superficial muscles**		**No muscles**	
Left	66.5	(18.16)	81.44	(21.18)	99.63	(1.48)
Right	74.43	(15.86)	85.98	(13.22)	98.19	(5.57)
Lumbar	85.03	(12.9)	94.87	(6.15)	99.96	(0.09)
**Classification 3**	**All muscles**		**Superficial muscles**		**No muscles**	
Left	61.2	(25.85)	71.32	(23.31)	87.32	(15.21)
Right	71.21	(17.1)	81.7	(13.61)	92.61	(3.95)
Lumbar	50.1	(29.4)	76.03	(23.86)	99.11	(2.01)
**Classification 4**	**All muscles**		**Superficial muscles**		**No muscles**	
Left	49.52	(25.84)	65.65	(23.99)	81.64	(23.23)
Right	65.06	(17.19)	81.4	(11.96)	97	(3.94)
Lumbar	47.2	(36.63)	83.03	(21.65)	87.44	(23.2)
**Classification 5**	**All muscles**		**Superficial muscles**		**No muscles**	
Left	68.9	(21.64)	68.8	(21.58)	91.05	(18.2)
Right	67.41	(16.44)	81.25	(11.74)	95.07	(6)
Lumbar	58.8	(37.53)	75.13	(24.12)	94.75	(7.73)

Average left, right and lumbar forces and their standard deviations applied over all Cobb angles across the five Classifications. Values are presented in N.

## Discussion

In this study, a musculoskeletal model of the spine was used to explore optimal combinations of muscle activation and applied external forces to reduce the magnitude of spinal curvature for different King’s classifications, across a range of Cobb angles. For Cobb angles lower than 20°, simulated muscle activation was more effective in reducing the objective function than application of external forces alone. This occurred over all classifications excluding classification 4, Figure 2. This observation is consistent with the conclusion of a previous simulation study that muscle activation can be equally effective as bracing at reducing the severity of a scoliotic curve [17], although it is noted that general external forces in place of a brace were simulated in the present study. The effectiveness of the simulated muscle activation at low Cobb angles is also consistent with clinical success reported in previous studies [5,6,12]. The simulation studies indicate reduced efficacy at higher Cobb angles which may account in part for the poor clinical outcomes of neuromuscular electrical stimulation at Cobb angles greater than 20 degrees [10].

Although the inclusion of deep muscles altered the results in certain cases, most notably Classifications 1 and 2, Figure 2(a,b), the results indicate that regardless of whether deep muscles were available to the optimizer, the same superficial muscle groups were targeted consistently across the majority of Cobb angles, Figure 3. However, the level of activation will vary across the superficial muscle groups. Therefore, there will not necessarily be a reduction in the number of superficial muscle groups activated when deep muscles are also targeted, but the same level of activation will not be required.

Earlier studies [5,12] have proposed that the optimal location for muscle stimulation for scoliosis correction is on the convex side of the curve. This held true for classifications 1–3 with the exception of the trapezius and pectorals where between 70% and 80% of the muscles activated were on the convex side of the thoracic curve, Figures 3 and 4. The serratus and intercostal muscle groups used the highest percentage of muscles on the convex side of the thoracic curve across all classifications, Figures 3 and 4. This agrees with clinical guidelines for delivering a straightening force to the spine by applying lateral electrical stimulation to the convex side of the curve [5].

For the majority of the curves, the activation of both the trapezius and the pectorals tended to occur on the concave side of the thoracic curves. A similar activation pattern also emerged for the single scoliotic configuration simulated in [17]. The correctional effects of the latissimus dorsi in the optimization are consistent with a previous study in which the latissimus dorsi in rabbits was activated to induce a concave curve on a straight spine [30]. To investigate this further, these muscle groups were isolated in the model, and similar activation patterns were applied. It was found that the latissimus dorsi induced a straightening force on the convex curve, while the trapezius and pectorals stabilized the spine. This suggests that stimulation on the concave side of the thoracic curve, in addition to the traditional stimulation on the convex side, may lead to better results and that the electrodes location should be chosen based on the type and location of the spinal curve.

The presence of the lumbar curves in classifications 1 and 2 did not result in muscle activation on their respective convex sides. The psoas and quadratus lumborum muscle groups were activated depending on the orientation of the lumbar curve in each classification. For classifications 1 and 2, the majority of muscle groups were activated on the concave side of the lumbar curve (convex side of the thoracic curve), Figure 4(a,b). Concave activation was also noted in the lumbar region of the curve in classification 4, Figure 4d. The muscles were activated in order to contract from the concave side to straighten the lumbar curvature. For classifications 3 and 5, the lumbar curve was small and the balance of the muscle activation for the psoas muscle group was almost even, Figure 4(c,e).

Similar patterns emerged when analysing the average force and the percentage of muscles activated across all Cobb angles and classifications. The highest average force applied to the model always occurred when muscles were excluded from the optimization, Figure 5. Although the reduction in objective function is numerically similar for many of the muscle and external force combinations, Figure 2, the composition of the results in terms of relative contribution of muscular and external force differed between combinations, Figures 5 and 6.

While computational models provide a means to examine complex *in vivo* systems, it is important to note the limitations inherent in the model and optimization. To perform a large number of optimizations efficiently, the direct stiffness method was chosen to simulate the relationship between the force applied to the model and the displacements within the model model. Another approach would be to use spring and damper systems to simulate the model stiffness and to include muscle models such as the Hill muscle model [31]. However, these methods would not be computationally efficient for the number of optimizations considered in this study. Since model displacements were small, soft tissue properties other than joint stiffness and intercostal tissue stiffness were not included, as they were assumed to be negligible. While the multifidus muscles are likely important in maintaining static equilibrium, their contribution in the present study was assumed to be negligible as the focus was the application of instantaneous corrective force to the model while in the prone position. The mechanical properties of the model were identical on the left and right sides in the normal upright position. It has been demonstrated by that side dominance affects muscle properties with regards to activation levels and fatigability [32]. This would also need to be considered in practice. The same objective function was utilised in all models to allow comparison across the conditions simulated. The sum of the squares of the vertebral distances was chosen, as it had been used in previous studies. Different objective functions such as combinations of individual vertebral rotations or curve areas could have also been used, and it is possible that tailoring different objective functions to each model would improve individual results. Kyphosis and lordosis, conditions often associated with scoliosis [6] were not examined in this study, as the main focus was the varying Cobb angle for the scoliotic curve. They could be accounted for by adjusting the objective function accordingly. Externally applied forces were not intended to represent any specific treatment directly but could be adjusted to be representative of brace forces or other therapeutic treatments involving external forces applied to the torso. Similarly, muscle activations elicited by the optimization were not necessarily representative of either electrical stimulation or physical therapy, and further adjustment would be necessary to represent a more realistic *in vivo* treatment. The results of this study show the instantaneous correction achieved by the optimizer. This provides an insight into which muscles should be activated to correct specific curves to achieve the best initial result but does not necessarily reflect the results that would be achieved in a long term *in vivo* treatment. The long-term sustainability of such treatments *in vivo* is therefore beyond the scope of this modelling study.

## Conclusion

This study suggests that consideration of the curve classification and Cobb angle is critical when assessing the potential for muscle activation for scoliosis correction. The results have indicated that superficial muscle activation on the convex side of the curve provides the best corrective results, supporting previous *in vivo* stimulation studies. They further suggest that additional benefit may be achieved through the simultaneous stimulation of muscle groups on the concave side of the curve.

The results suggest the potential for a hybrid treatment at low Cobb angles involving muscle activation, possibly achieved through electrical stimulation, and applied external forces which may offer a more comfortable and effective clinical alternative to bracing alone. The identification of muscles capable of applying a corrective force to scoliotic curves could also be useful in both postural control devices and therapeutic exercises. Further studies are necessary to investigate this potential before recommending any clinical application, most notably *in vivo* studies and an investigation of the sustainability resulting from these treatments.

## Competing interests

The authors declare that they have no competing interests.

## Authors’ contributions

MC participated in the study design and carried out the model simulations and optimizations. ML participated in the study design and helped to draft the manuscript. All authors read and approved the final manuscript.
